# A comprehensive DNA barcoding of Indian freshwater fishes of the Indus River system, Beas

**DOI:** 10.1038/s41598-024-52519-0

**Published:** 2024-02-02

**Authors:** Sonakshi Modeel, Ram Krishan Negi, Monika Sharma, Padma Dolkar, Sheetal Yadav, Sneha Siwach, Pankaj Yadav, Tarana Negi

**Affiliations:** 1 Fish Molecular Biology Lab, Department of Zoology, University of Delhi, North Campus, Delhi, 110007 India; 2 Department of Zoology, Govt. College Dujana, District Jhajjar, Beri, Haryana India

**Keywords:** Biotechnology, Animal biotechnology, Genomics

## Abstract

The Beas River is one of the important rivers of the Indus River system located in Himachal Pradesh, India, that harbors a diverse range of freshwater fish species. The present study employed COI gene to investigate the ichthyofaunal diversity of river Beas. Through the sequencing of 203 specimens from Beas River, we identified 43 species, belonging to 31 genera, 16 families, and 10 orders. To analyze the genetic divergence and phylogeny of identified species, 485 sequences of Indian origin were retrieved from BOLD, resulting in a dataset of 688 sequences. Our findings consistently revealed a hierarchical increase in the mean K2P genetic divergence within species (0.80%), genus (9.06%), and families (15.35%). Automated Barcode Gap discovery, Neighbour Joining, and Bayesian inference consensus tree methodologies were employed to determine the putative species and their phylogeny, successfully delimiting most of the species with only a few exceptions. The results unveiled six species exhibiting high intra-species divergence (> 2%), suggesting the presence of sibling species and falsely identified sequences on online databases. The present study established the first DNA barcoding-based inventory of freshwater fish species in the Beas River providing comprehensive insights into economically exploited endangered and vulnerable species. In order to ensure the sustainable use of aquatic resources in the Beas River, we recommend the implementation of species measures to protect biodiversity and genetic resources.

## Introduction

Accurate species identification plays a crucial role in understanding how evolutionary history, biodiversity patterns, and ecosystems change over time. Precise identification enables the detection of invasive, elusive, endangered, or threatened species, and helps reduce various ecosystem risks by facilitating the habitat for conservation. To effectively manage the genetic resources of fish species sustainably, a comprehensive understanding of the taxonomy and systematics of fish species is very essential. DNA-based identification techniques have greatly improved the reliability of species identification, particularly for cryptic species and larval fishes^1,2^. DNA barcoding utilizes a standardized gene called DNA barcode, which offers a reliable method for species identification while providing valuable insights into evolutionary relationships^3,4^. The flanking regions of barcode sequences should exhibit minimal sequence variations for efficient amplification, and the mutation rate of the genes used for taxonomic identification of closely related species should be below 2%^5^. The mitochondrial cytochrome c oxidase subunit-I gene (COI) is the preferred DNA barcode due to its ability to provide a wide range of phylogenetic signals, robust primers, and a high occurrence of base substitutions in third-position nucleotides, resulting in accelerated molecular evolution^4^. The COI gene sequence finds extensive applications in various fields, such as forensic science, food authentication, and biotechnology-based industries, owing to its prevalence in mitochondrial DNA^2^. COI has revealed divergent patterns of evolution and possible hidden variations from the present taxonomic classifications^6–10^. Understanding the processes of community construction can be gained from well-resolved phylogenies produced from DNA barcodes when combined with functional characteristic data of the same species^11^. Reconstructing the phylogenetic relationships from large groups of species is crucial for taxonomic studies to address various ecological problems. One of the current problems in taxonomic identification is the presence of cryptic species, which refers to species with minor physical variability but significant genetic variations^12^. Cryptic species might result by means of parallel evolution from recent divergence in closely related taxa or distant taxa in a comparatively short amount of time^13^. To represent genetic differentiation within a species, cryptic species sensu stricto and sensu lato terms are also employed^14–16^. The former represent genetically distinct species that have been shown to lack diagnostic morphology, while the latter represent genetically distinct species that are hard to visually identify but possess some distinctive phenotypic trait(s)^13,17^. The existence of cryptic species has been effectively established by a number of studies that have employed DNA barcoding to taxonomically identify fish species^18–24^. The COI gene also serves as a valuable genetic marker to evaluate population genetic structure of metazoans through the identification of distinct haplotypes across various geographical regions^25–29^.

India is boasted for its a rich diversity of aquatic species, including over 1045 freshwater fish species^30^. The Beas River, spanning 470 km through the Himalayan region of Northern India, represents a significant aquatic resource. Originating from Rohtang Pass in Himachal Pradesh and merging with Satluj River at Harike Wetlands in Punjab, the Beas River has undergone substantial modifications that have impacted ecology and fish diversity. A report from the Central Inland Fisheries Research Institute, Barrackpore has identified a total of 54 fish species in the river Beas^31^. According to survey, Indian major carp comprised 28.28% of fishery in the Beas, with minor carps ranking second at 22.44% followed by common carp at 22.02%, and other species at 17.75%. Catfish, on the other hand, were the least prevalent, accounting for 8.54% of the fishery. Kumar^32,33^ found only six fish species from the upstream of the Beas River, indicating poor faunal biodiversity. Harike Wetlands, located at the confluence of the Beas and Satluj river systems and designated as one of India's Ramsar Sites (internationally recognized wetlands under the Ramsar Convention), showcase a diverse range of fish species. In 2009, Dua^34^ identified 61 fish species in the Harike Wetlands, while Kour^35^ reported 37 species in 2017. These findings indicate the highest number of species from the Cyprinidae family, encompassing fish species from both the River Beas and Satluj. While limited research has been conducted on the ichthyofaunal diversity of the Beas River system in the past decade, only a handful of ichthyologists have employed morphological approaches to investigate fish diversity in the system which has resulted in a deficiency in our comprehension of the fish fauna in the region.

The primary objective of the present study was to establish a DNA barcoding-based inventory of freshwater fish species in Beas River and gain a deeper understanding of their genetic variation and distribution pattern. The results of the current study offer significant data that can enhance monitoring and conservation efforts aimed at efficient management of the Beas River. The study incorporates online available DNA barcodes of Indian origin to analyze and authenticate DNA barcoding and explore their genetic diversity. The current study provides new insights into the taxonomic affiliation and divergent patterns of evolution through the application of the COI gene to examine the haplotype diversity and population genetic structure of multiple fish species.

## Results

### DNA barcoding of fishes of Beas River

**Figure 1:**
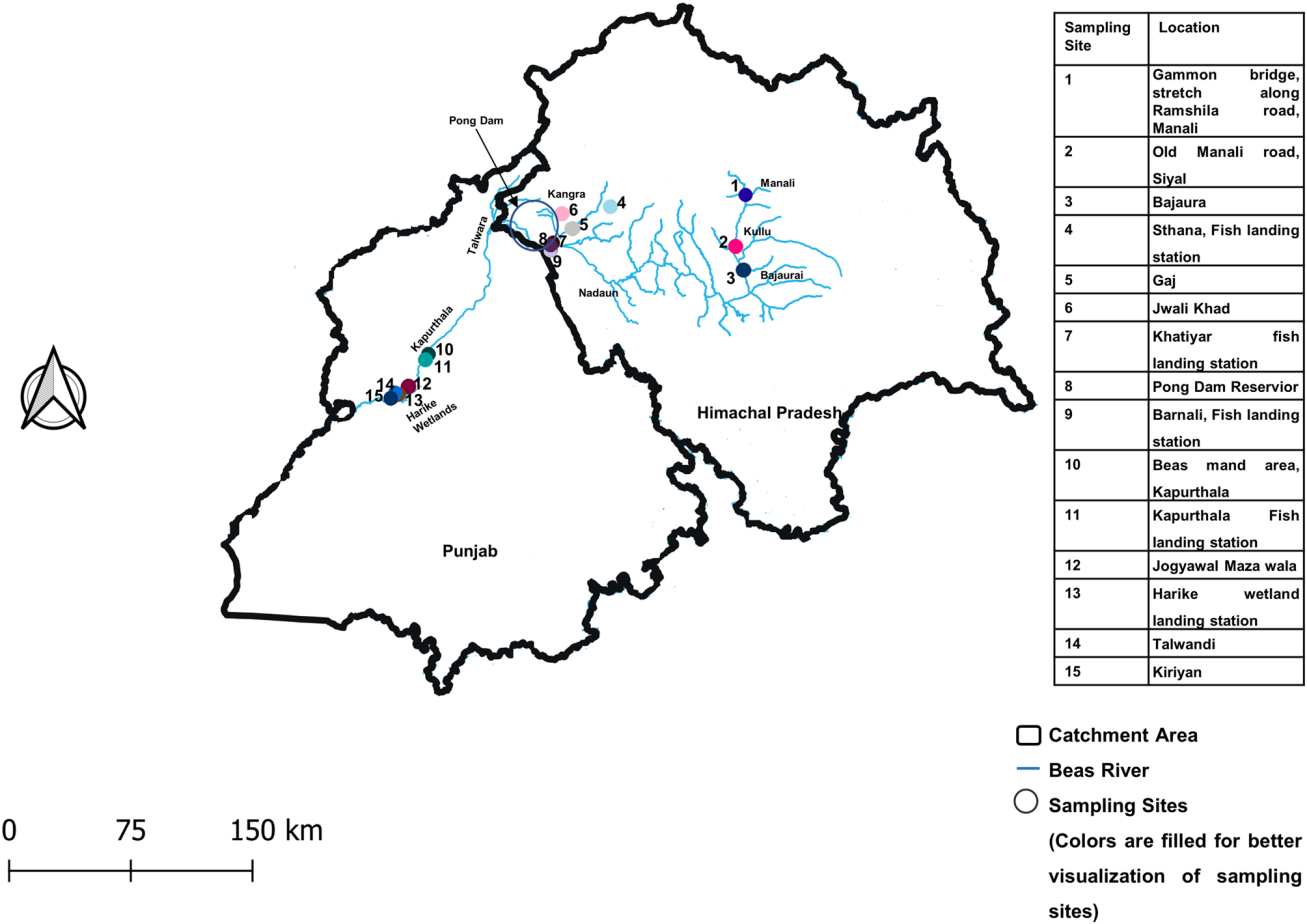
Map representing sampling sites in River Beas and its tributaries in Himachal Pradesh and Punjab, India. Created in QGIS v 3.26.2 (https://www.qgis.org/en/site/).

A total of 320 fish specimens were collected from 15 different sites, including major commercial fish landing stations on Beas River (Fig. 1). Out of these, sequences were successfully generated for 203 specimens, resulting in 100% amplification rate of COI barcode. All the amplified sequences represented functional COI sequences of length > 600 bp without any deletion, insertion, or stop codon. Similarity search was performed using the BOLD identification engine and NCBI nucleotide blast. The similarity percentage for all the sequences ranged from 98 to 100%. The overall GC content was 45.49% (SE = 0.10). Table 1 provides the nucleotide composition, GC content, and GC% at codon positions 1, 2, and 3 for all orders. The dataset of 203 sequences represented 43 species, 31 genera, 16 families, and 10 orders. The order Cypriniformes was the most diverse, comprising 135 specimens (66.5%) followed by Siluriformes (14.7%), Anabantiformes (5.4%), Salmoniformes (3.4%), Ovalentaria (3.4%), Beloniformes (1.9%), Clupeiformes (1.9%), Osteoglossiformes (1.4%), Cichliformes (0.4%), and Synbranchiformes (0.4%). The Cyprinidae family was most abundant comprising 21 species, followed by Danionidae with 5 species. The Bagridae, Channidae, and Siluridae families each were represented by 2 species, while the Ailidae, Ambassidae, Belonidae, Cichlidae, Clupeidae, Heteropneustidae, Mastacembelidae, Nandidae, Notopteridae, Salmonidae, and Sisoridae, families were represented by 1 species each. The classification, number of specimens barcoded, and the IUCN status of the fish species are presented in the Table 2.

**Table 1:** The overall and order-wise nucleotide composition, GC content, and GC% at codon positions 1, 2, and 3 of fishes of River Beas.

Nucleotide (%)								
	G	C	A	T	GC	GC1	GC2	GC3
Overall	18.07	27.40	25.78	28.76	45.47	56.33	43.58	36.49
Anabantiformes	18.69	25.92	24.08	31.32	44.61	56.21	43.06	34.54
Beloniformes	17.16	26.40	25.04	31.41	43.55	54.01	43.44	33.18
Cichliformes	17.29	29.62	24.51	28.57	46.92	57.01	42.79	40.99
Clupeiformes	18.72	28.22	23.50	29.56	46.94	56.74	43.32	40.78
Cypriniformes	18.01	27.44	26.22	28.32	45.46	56.71	43.80	35.87
Osteoglossoformes	18.42	27.42	24.81	29.36	45.83	56.38	43.06	38.08
Ovalentaria	18.56	26.45	23.55	31.44	45.01	57.25	43.42	34.36
Salmoniformes	18.40	29.46	23.00	29.14	47.86	55.46	43.53	44.60
Siluriformes	18.16	27.46	25.06	29.32	45.63	55.76	43.24	37.89
Synbranchiformes	18.31	29.37	23.23	29.09	47.68	55.54	43.49	44.01

**Table 2:** Classification, number of specimens barcoded, and the IUCN status of the fish species of River Beas, India using DNA barcoding.

Order	Family	Genus	Species	Specimen number	IUCN status
Anabantiformes	Channidae	*Channa*	*Channa punctata*	3	Least concern
			*Channa marulius*	4	Least concern
	Nandidae	*Nandus*	*Nandus nandus*	4	Least concern
Beloniformes	Belonidae	*Xenentodon*	*Xenentodon cancila*	4	Least concern
Cichliformes	Cichlidae	*Oreochromis*	*Oreochromis niloticus*	1	Least concern
Clupeiformes	Clupeidae	*Gudusia*	*Gudusia chapra*	4	Least concern
Cypriniformes	Cyprinidae	*Chagunius*	*Chagunius chagunio*	1	Least concern
		*Labeo*	*Labeo gonius*	9	Least concern
			*Labeo calbasu*	5	Least concern
			*Labeo catla*	15	Least concern
			*Labeo bata*	3	Least concern
			*Labeo boggut*	2	Least concern
			*Labeo rohita*	21	Least concern
		*Cyprinus*	*Cyprinus carpio*	4	Vulnerable
		*Cirrhinus*	*Cirrhinus mrigala*	4	Least concern
			*Cirrhinus reba*	1	Least concern
			*Cirrhinus cirrhosus*	1	Vulnerable
		*Systomus*	*Systomus sarana*	2	Least Concern
		*Tor*	*Tor tor*	3	Data deficient
			*Tor putitora*	20	Endangered
		*Bangana*	*Bangana dero*	1	Least concern
		*Tariqilabeo*	*Tariqilabeo adiscus*	4	Data deficient
			*Tariqilabeo latius*	2	Least concern
		*Schizothorax*	*Schizothorax plagiostomus*	7	Vulnerable
			*Schizothorax richardsonii*	1	Vulnerable
		*Osteobrama*	*Osteobrama cotio*	2	Least concern
		*Pethia*	*Pethia conchonius*	4	Least concern
	Danionidae	*Esomus*	*Esomus danrica*	3	Least concern
		*Cabdio*	*Cabdio morar*	4	Least concern
		*Barilius*	*Barilius vagra*	1	Least concern
		*Salmostoma*	*Salmostoma phulo*	11	Least concern
			*Salmostoma bacaila*	4	Least concern
Osteoglossoformes	Notopteridae	*Notopterus*	*Notopterus notopterus*	3	Least concern
Ovalentaria	Ambassidae	*Chanda*	*Chanda nama*	7	Least concern
Salmoniformes	Salmonidae	*Salmo*	*Salmo trutta fario*	7	Least concern
Siluriformes	Ailiidae	*Clupisoma*	*Clupisoma prateri*	1	Least concern
	Bagridae	*Rita*	*Rita rita*	1	Least concern
		*Sperata*	*Sperata seenghala*	15	Least concern
	Siluridae	*Wallago*	*Wallago attu*	3	Vulnerable
		*Ompok*	*Ompok pabo*	2	Near threatened
	Sisoridae	*Bagarius*	*Bagarius bagarius*	1	Vulnerable
	Heteropneustidae	*Heteropneustes*	*Heteropneustes fossilis*	7	Least concern
Synbranchiformes	Mastacembelidae	*Mastacembelus*	*Mastacembelus aramatus*	1	Least concern

### Sequence composition and genetic divergence

To infer the genetic divergence, the 203 sequences obtained from Beas River were combined with 485 records from the BOLD database (Supplementary Table S1). The mean K2P genetic divergence within species (0.80%), genus (9.06%), and family (15.35%) showed a consistent hierarchical increase in genetic diversity with rising taxonomic levels (Table 3). The average congeneric divergence was 11 times higher than the conspecific divergence. The K2P genetic divergence was calculated within and between families. Family Danionidae and Cyprinidae showed the highest genetic divergence (0.21 ± 0.02 and 0.14 ± 0.01 respectively), whereas Sisoridae showed the lowest (0.001 ± 0.0005). Cichlidae, Clupeidae, and Salmonidae showed no genetic divergence due to the presence of a single species. Estimates of evolutionary divergence over sequence pairs between families showed the highest divergence between Bagridae and Nandidae (0.41 ± 0.05), while the minimum was observed in Heteropneustidae and Ailiidae (0.19 ± 0.02). K2P genetic divergence was also calculated between and within all the species. The inter-species genetic divergence was also calculated, revealing the highest K2P genetic divergence (44%) between *Nandus nandus* and *Sperata seenghala*, the lowest (0.4%) between *Bangana dero* and *Labeo boggut,* and negligible between *Schizothorax plagiostomus* and *Schizothorax richardsonii*.

**Table 3:** Summary of sequence divergence (K2P) distribution for different taxonomic levels.

	n	Taxa	Comparisons	Min Dist(%)	Mean Dist(%) and SE	Max Dist(%)
Within species	688	43	7701	0	0.80 ± 0.00	15.45
Within genus	326	7	12,360	0	9.06 ± 0.00	19.49
Within family	469	2	77,264	0	15.35 ± 0.00	24.72

### Phylogenetic analysis and species delimitation

**Figure 2:**
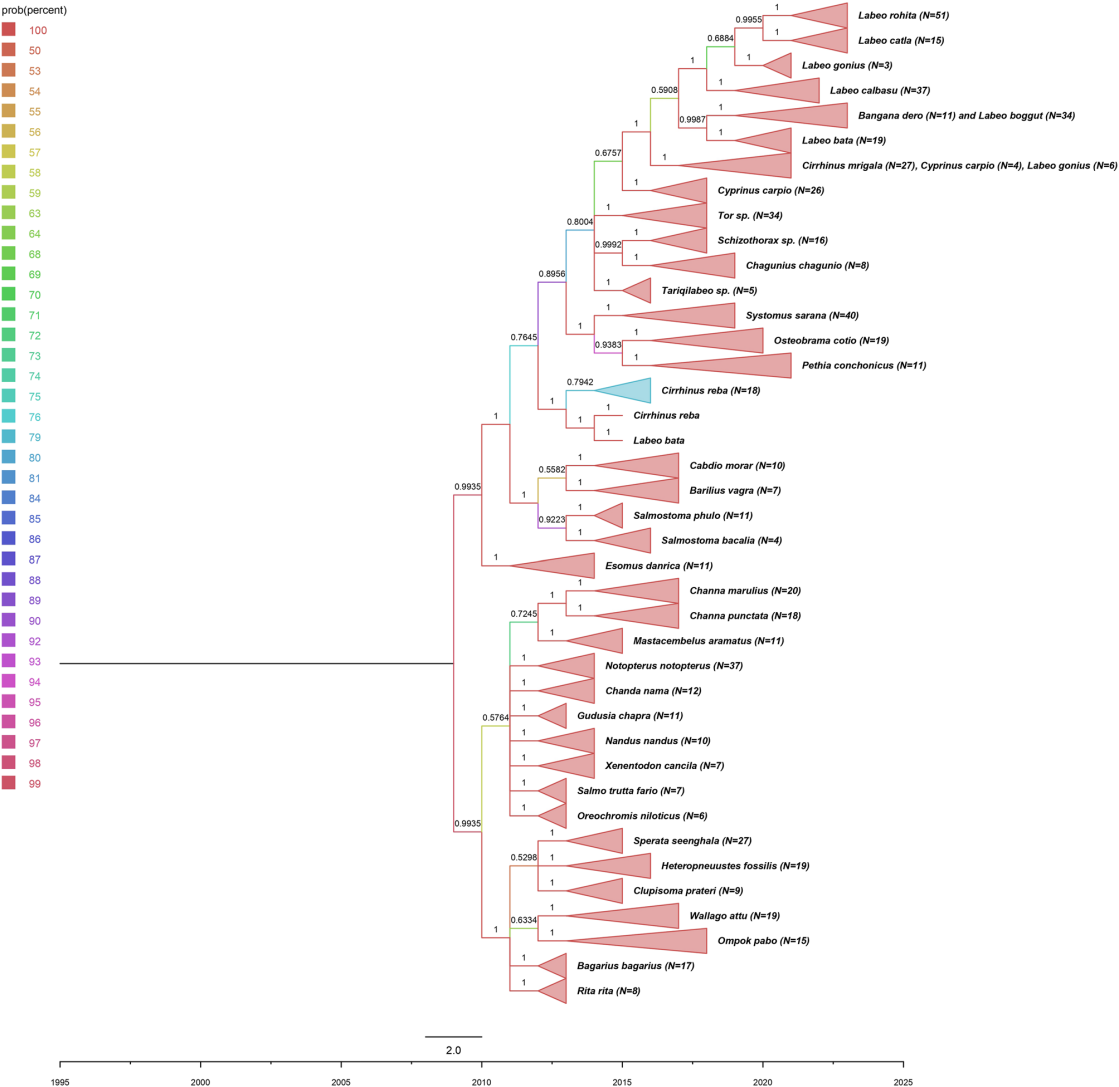
Bayesian inference (BI) consensus tree based on 688 COI barcodes under the GTR + I + Γ reversible evolution model. The number on branches depicts probabilities and the color illustrates probability percentage.

The ABGD analysis of 688 COI sequences identified a cluster of 8 molecularly defined operational taxonomic units (MOTUs). The prior maximal distance (p) of 0.0077 was sufficient to distinguish fish species in present study. The overall dataset of 688 sequences belonging to 43 species was clustered into 56 groups (Supplementary Fig. S1). Most of the species were clearly delimited into putative species, while *Schizothorax* sp. (*S. richardsonii* and *S. plagiostomus*) and *Tariqilabeo* sp. (*T. adiscus* and *T. latius*) could not be delimited properly. A few sequences belonging to species *Cirrhinus cirrhosis*, *Bangana dero*, *Tor tor*, *Cirrhinus reba*, and *Labeo bata* were grouped together with their respective nearest neighbor (Supplementary Table S2). Some groups in the partition were represented by single sequences, which indicate geographically isolated or genetically distinct haplotype of their respective species. To enhance species delimitation, Neighbor-joining (Supplementary Fig. S2) and Bayesian Inference (BI) (Fig. 2) was also conducted using the complete dataset of 688 sequences and the resulting trees showed consistent findings with ABGD species delimitation.

### Genetic diversity and haplotype networks of specific taxa

**Figure 3:**
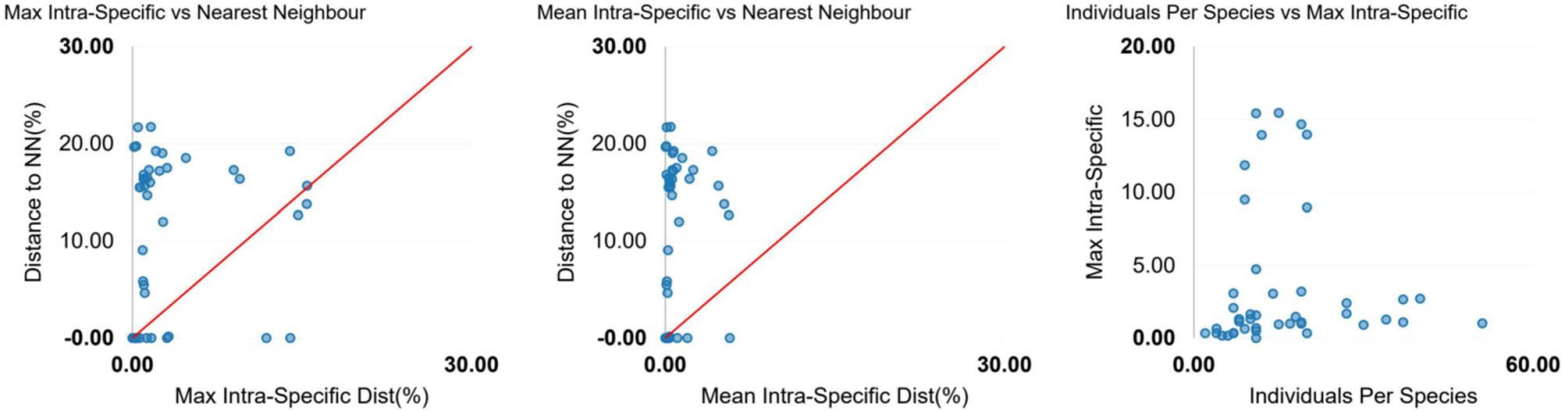
Barcode gap analysis: (**a**) overlap of the max intra-species distances vs the inter-species (nearest neighbor) distances; (**b**) overlap of the mean intra-species distances vs the inter-species (nearest neighbor) distances; (**c**) the number of individuals in each species against their max intra-species distances, as a test for sampling bias.

**Figure 4:**
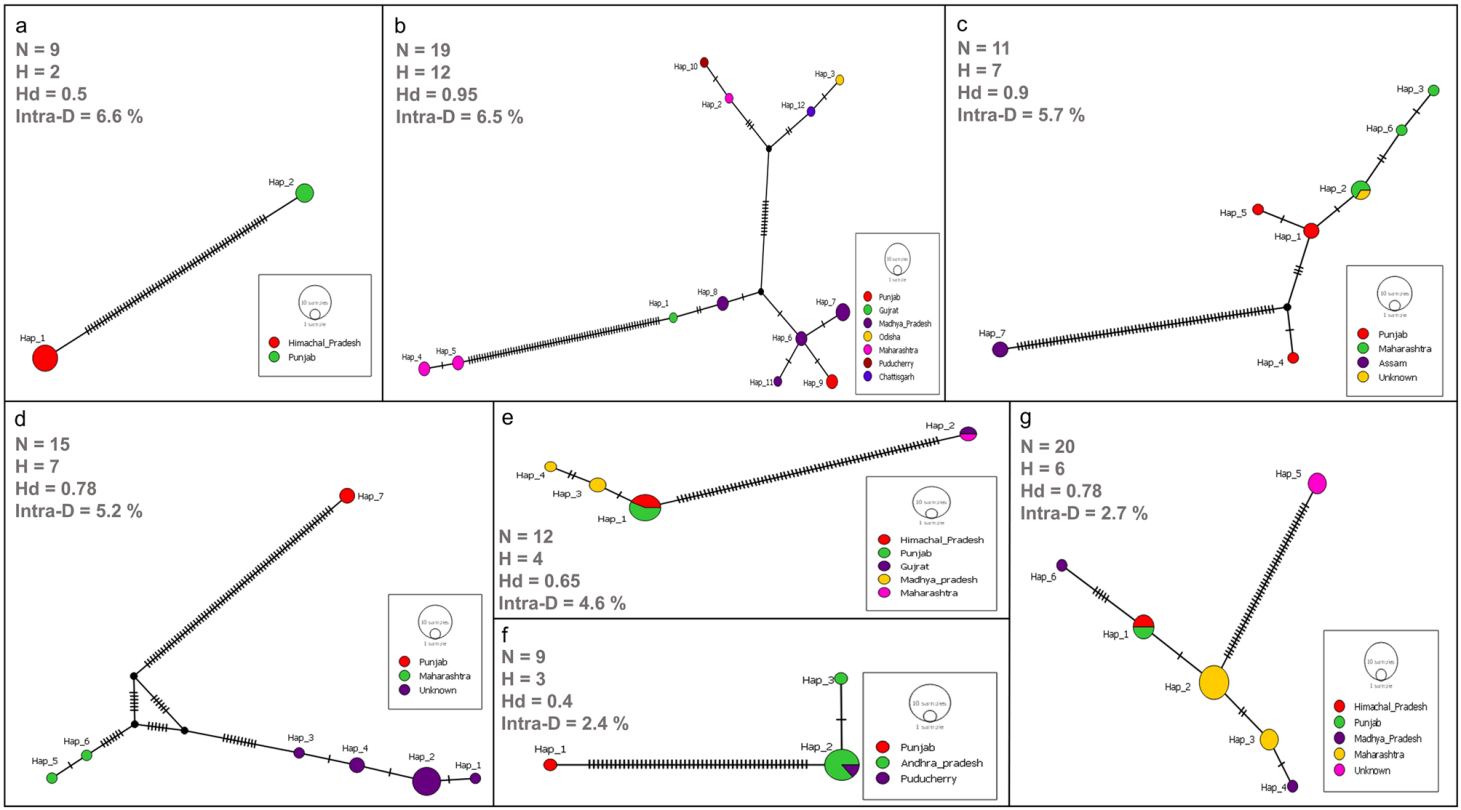
Median-joining networks and haplotype diversity indices of species with intra-species diversity > 2%; (**a**) *Labeo gonius*, (**b**) *Osteobrama cotio*, (**c**) *Pethia conchonius*, (**d**) *Ompok pabo*, (**e**) *Chanda nama*, (**f**) *Clupisoma prateri*, and (**g**) *Channa marulius*. The size of the circle represents the relative frequency of the associated haplotype over the entire dataset of respective species, and the colors represent the related state/province in India. The number of mutational changes between two haplotypes is represented by the black lines on the branches. Values for number of sequences (N), haplotype diversity (Hd), number of haplotypes (H), and Intra-species diversity (Intra-D) are given along with each haplotype network.

The intra-species diversity observed from all the species ranged from 0 to 6%. The highest intra-species diversity was observed among *Labeo gonius* sequences (6.6%) and the lowest were observed in *Systomus sarana* (1.2%), *Barilius vagra* (0.9%) and *Tor tor* (0.9%) (Fig. 3). Significantly high intra-species diversity (> 2%) was observed in *L. gonius* (6.6%), *O. cotio* (6.5%), *P. conchonius* (5.7%)*, O. pabo* (5.2%)*, C. nama* (4.6%)*, C. marulius* (2.7%) and *C. prateri* (2.4%). Distinct clusters of haplotypes can be observed in median-joining haplotype network analysis (Fig. 4). All the results showed similar partitions in ABGD species delimitation, indicating the possibility of presence of geographically divergent or sibling species. Moreover, genus *Channa* (12%), *Cirrhinus* (9%), and *Labeo* (7%) showed notable congeneric divergence.

**Figure 5:**
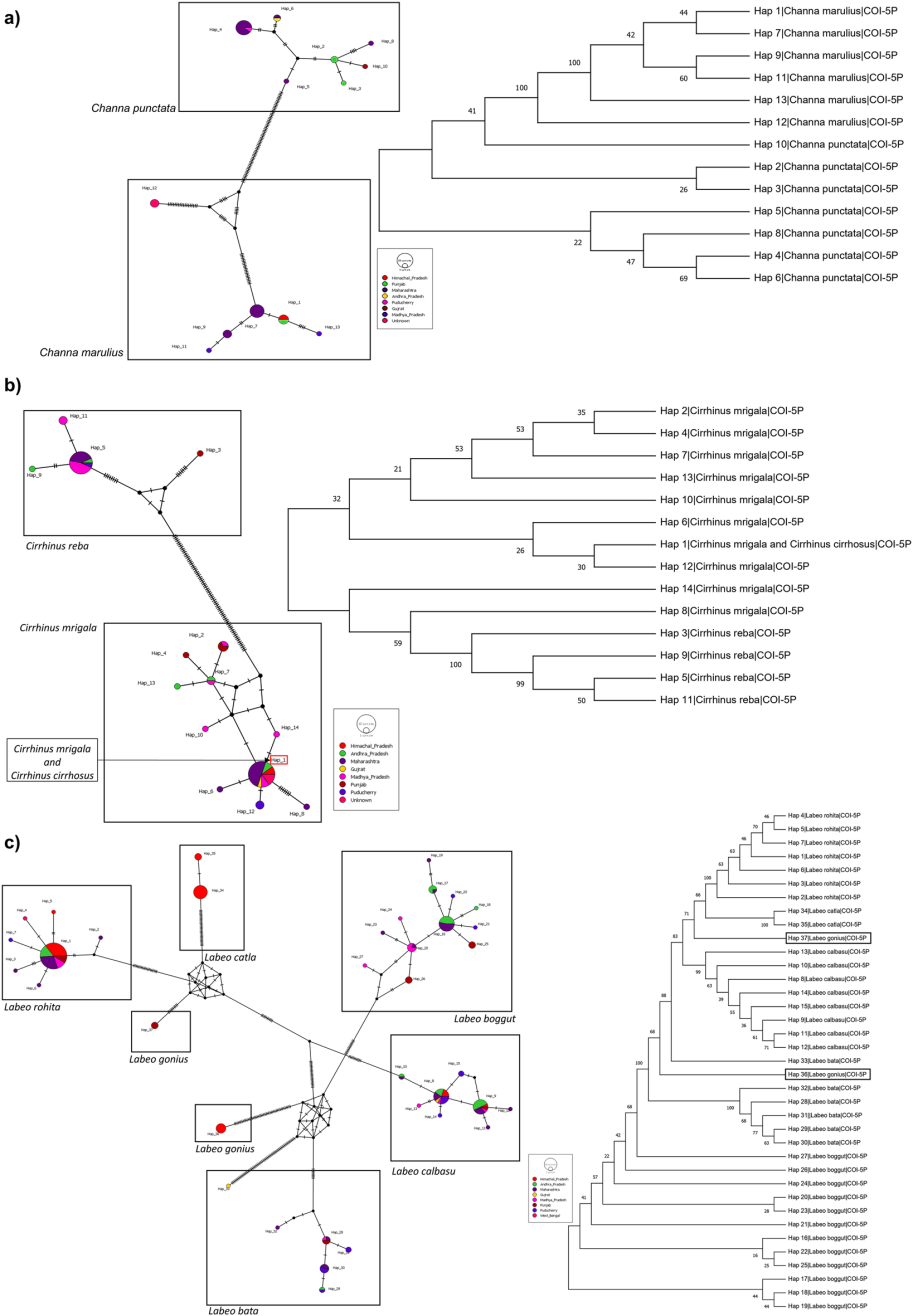
Neighbour-joining (NJ) phylogenetic tree and median-joining haplotype network showing distinct lineages of species from (**a**) genus *Channa* (*C. punctata* and *C. marulius*) haplotypes, (**b**) genus *Cirrhinus* (*C. reba*, *C. mrigala* and *C. cirrhosus*) haplotypes, (**c**) genus *Labeo* (*L. rohita*, *L. catla*, *L. boggut*, *L. bata*, *L. gonius* and *L. calbasu*) haplotypes. The NJ phylogenetic tree is constructed with a bootstrap value of 1000 using the MEGAX software package. The size of the circle in haplotype networks represents the relative frequency of the associated haplotype over the entire dataset, and the colors represent the related state/province in India. The number of mutational changes between two haplotypes is represented by the black lines on the branches.

(a) *Channa* Species: The genus *Channa* comprised a total of 38 sequences belonging to *C. marulius* (20) and *C. punctata* (18). Genetic divergence among genus *Channa* was highest (12%) among all other genera. *C. marulius* showed intra-species diversity of 2.7%, haplotype diversity (Hd) of 0.78 and 6 haplotypes (H), whereas *C. punctata* revealed lower intra-species diversity (0.7%), comparatively lower haplotype diversity (0.69) and higher number of haplotypes (7). The inter-species divergence between *C. marulius* and *C. punctata* was 2.9%. The NJ tree of haplotypes and median-joining network of genus *Channa* shows haplotype 12 is separated by haplotype 7 by 44 mutations, which reveals highly divergent population of *C. marulius* (Fig. 5a)*.* The ABGD species delimitation also reveals that all the sequences of haplotype 12 are forming a separate group at optimal threshold (P = 0.0077), suggesting the formation of a new putative species of *Channa*. (b) *Cirrhinus* Species: In the present study, there were a total of 51 sequences from the *Cirrhinus* genus, including sequences from *C. reba* (19), *C. mrigala* (27), and *C. cirrhosis* (5) revealing 9% of genetic divergence within genus. The K2P divergence was 19% between both *C. reba* and *C. mrigala*; and between *C. reba* and *C*. *cirrhosis*. The K2P divergence was negligible between *C. mrigala* and *C. cirrhosis*. C*. reba* and *C. mrigala* had nearly equal intra-species divergence (0.35%), however *C. mrigal* (H = 10; Hd = 0.68) had a greater number of haplotypes and higher haplotype diversity than *C. reba* (H = 4; Hd = 0.38). On the other hand, *C. cirrhosis* showed only one haplotype and no intra-species diversity. Two distinct sets of haplotypes can be seen in the median joining network of haplotypes and NJ tree, one belonging to *C. reba* (Hap 3, 5, 9, and 11), and the other to *C. mrigala* and *C. cirrhosis* (Hap 1, 2, 4, 6, 7, 8, 10, 12, 13, and 14) (Fig. 5b). Haplotype 1 consisted of 15 sequences of *C. mrigala* and 5 sequences of *C. reba*, which demonstrated the possibility of falsely identified lineages on online database or presence of introgressive hybridization between them. (c) *Labeo* Species: A total of 172 sequences constituting five species; *L. rohita* (51)*, L. calbasu* (37)*, L. boggut* (34)*, L. bata* (20), *L. catla* (15)*,* and *L. gonius* (9)*.* The K2P genetic divergence observed within genus was 7%. The inter-species diversity between species of genus *Labeo* was highest between *L. catla* and *L. gonius* (12.5%) and lowest between *L. bata* and *L. boggut* (5%). The highest K2P divergence within species was observed in *L. boggut* (0.3%) and highest in *L. gonius* (6%). *L. gonius* was the only species in genus *Labeo* showing more than 2% intra-species divergence indicating presence of highly diverged sibling species. All the sequences of *L. gonius* were double checked for similarity percentage of the sequence on NCBI blast and BOLD IDs databases, which revealed 100% similarity with* L. gonius*. The number of haplotypes (H) and haplotype diversity (Hd) were also calculated, revealing the *L. gonius* haplotypes (Hap 36 and 37), which are substantially divergent (Fig. 5 c). Higher intra-species divergence (1.9%) and haplotype diversity of 0.76 (H = 7) were also observed in *L. bata*. Haplotype 33 of *L. bata* population showed a highly divergent group from the remaining cluster of haplotypes (Hap 28, 29, 30, 31, 32), possibly due to genetically isolated or geographically distant population.

## Discussion

DNA barcoding is a useful tool for identifying species by creating barcodes from different geographical regions. Various factors such as genetic drift, ecological factors, and developmental flexibility can lead to population-level diversification and speciation, resulting in variations in genotypic and phenotypic features of species across geographic regions^36^. In this study, we contributed a novel perspective on fish biodiversity and conservation by presenting the first molecular-based assessment of the fish fauna in Beas River. The study encompassed the establishment of a reference library comprising 203 DNA barcodes from 43 different species belonging to 10 orders, with Cypriniformes exhibiting the highest diversity, followed by Siluriformes. Among the 43 species investigated, 27 were previously documented in the Beas River^32,33^. The current study has documented 16 species for the first time including *Bangana dero, Barilius vagra, Cabdio morar, Chagunius chagunio, Chanda nama, Cirrhinus cirrhosis, Clupisoma prateri, Esomus danrica, Labeo boggut, Oreochromis niloticus, Salmostoma bacaila, Salmostoma phulo, Schizothorax plagiostomus, Tariqilabeo adiscus, Tariqilabeo latius, and Tor tor*. Although some of these species have been previously reported in studies based on the Harike wetlands, which encompass both the Satluj River and the Beas River, it may not be accurate to directly compare these findings when estimating the ichthyofaunal diversity specifically within the Beas River^34,35,37^. The base composition analysis of the 203 sequences from the Beas River indicated a richness in AT content (54.54%). A lower GC composition (45.47%) and G (18.07%) were observed, characteristics of mitochondrial DNA in fishes. The first codon GC content was notably higher than the other two positions, with the base usage bias at the third codon position being the lowest. This pattern aligns with observation in other studies where the third codon position exhibited a clear anti-G bias^38–41^.

To assess genetic divergence and established phylogenetic relationships on a wider scale, we aggregated 485 sequences from Indian origin on BOLD database with sequences from the Beas River. The K2P genetic divergence within the dataset of 688 sequences was 0.8% within species, escalating to 9.06% within genus and 15.35% within family. This hierarchical increase in divergence with the progression of taxonomic levels has been also observed in previous studies on fish populations including Australian marine fish (0.39%, 9.93%, 15.46%)^7^, Canadian freshwater fishes (0.27%, 8.37%, 15.38%)^42^, Indian Marine fishes (0.30%, 6.60%, 9.91%) ^42^, Narmada River, India (0.36%, 12.29%, 17.87%)^43^, Indian freshwater fishes (0.40%, 9.60%, 13.10%)^39^, Indo-Myanmar fishes (0.42%, 10.19% and 12.77%)^39^, and Ranganadi River, India (0.23%, 11.31%, 26.25%) ^44^. The consistent hierarchical increase in divergence across diverse studies and regions among fish populations likely reflects the accumulation of genetic differences and adaptive divergence over time. This pattern carries significant ecological and evolutionary implications, underscoring the importance of tailored conservation strategies to preserve specialized genetic adaptations. It also highlights the remarkable biodiversity within fish families that warrants recognition and protection. This consistent pattern observed across various regions suggests the fundamental nature of this phenomenon. Further research is needed to understand the genetic mechanisms behind this consistent pattern that will help better understand the evolution of species and the diversity of their genes across the different taxonomic levels.

Numerous studies have used the ABGD tool for the construction of phylogenetic trees and species delimitation. ABGD clusters sequences into candidate species based on the pairwise distances and barcode gap^10,43,45,46^. In this study, ABGD analysis successfully differentiated all species except for *Schizothorax* (*S. richardsonii* and *S. plagiostomus*), *Tariqilabeo* (*T. adiscus* and *T. latius*) and a few sequences of *Cirrhinus cirrhosis*, *Bangana dero*, *Tor tor*, *Cirrhinus reba*, and *Labeo bata*. These finding were corroborated through phylogenetic reconstruction using the Neighbour joining and Bayesian inference (BI) tree. The utilization of native populations in hatcheries for artificial breeding and the potential interbreeding between escaped farmed fish and wild types may have detrimental effects on the genetic integrity of the fish species^17,25^. The presence of introgressive hybridization is indicated by the shared mitochondrial lineage and low intra-species divergence values, particularly between *S. richardsonii* and *S. plagiostomus* as well as *T. adiscus* and *T. latius*. Mitochondrial introgression occurs when a species possesses two mitochondrial lineages, one from its own mitochondria and the other acquired from another species^26^. Introgression and hybridization associated with inter-species gene flow, enable the integration of novel genetic information into the parent species' genome, resulting in species harboring both mitochondrial lineages^27^. Akhtar ^47^ revealed genetic divergence of 0.009% between *S. plagiostomus, S. esocinus, S. progastus,* and *S. niger* suggesting the possibility of ancestral polymorphism and introgressive hybridization. The sequences for the genus *Schizothorax* (*S. richardsonii* and *S. plagiostomus*) in the present investigations were restricted to Beas River due to the absence of trace files for remaining sequences in the BOLD database. Reconstructing phylogenetic relationships becomes more challenging when there is ancestral hybridization and polyploidization, causing limited gene transmission between different populations or species^48,49^. Furthermore, comprehending the mitochondrial complexity of such species proves challenging due to limited sample size and geographically confined locations. The present study also showed *Labeo boggut* and *Bangana dero*, both belonging to family Cyprinidae and sub-family Labeoninae, in a single monophyletic clade in COI lineage. This observation aligns with previous studies where *Labeo* sp. and *Bangana* sp. formed a unified clade^50,51^. Yang^31^ demonstrated that *B. dero* formed a distinct lineage with *L.* *bata*, *L. boggut*, and three additional forms, naming the clade “*Bangana sensu stricto*”. The studies on the molecular phylogeny of genus *Bangana* are in its early stages and, its systematic status remains debatable^32^.

Intra-species genetic divergence exceeding 2% was observed in *Labeo gonius*, *Osteobrama cotio*, *Pethia conchonius*, *Ompok pabo*, *Chanda nama*, *Channa marulius*, and *Clupisoma prateri*. *O. cotio* exhibited a 6.5% divergence, supported by a previous study on the species, based on COI and 16srRNA gene sequences from rivers in India and Bangladesh^52^, suggesting genetic differentiation of different populations of *Osteobrama* and the possible existence of a new putative species named *O. serrata*. High intra-species divergence within species suggests the potential presence of geographically divergent or sibling species formed due to adaptation and high evolutionary potential of the species^53^. The genetic divergence values of *P. conchonius* in Ganga and Yamuna rivers of Uttrakhand, India, as reported by Joshi^54^ did not reveal higher values compared to the current study. Negi ^55^ reported a falsely identified sequence for *Puntius chola* and noted close proximity between sequences of *Puntius terro* and *Pethia conchonius*. Haplotype diversity and the number of haplotypes were also investigated for species with high genetic divergence; *O. cotio, P. conchonius, O. pabo* and *C. marulius*, showing high haplotype diversity, and *L. gonius*, *C. nama*, and *C. prateri* exhibiting moderate diversity. All these highly diverged species exhibit at least one haplotype with significant mutations, distinctly separated by clusters of other haplotypes. Sequences from these unique haplotypes form distinct group in the ABGD species delimitation analysis, supporting the potential formation of new putative species. Following the criteria outlined by Hebert, and Stoeckle^56^, if the genetic divergence between two specimens of a supposedly single species is greater than ten times the average divergence within that group, it is likely that one of them represents a new putative species. When proposing a species as a potentially new species, it is essential to conduct thorough examination of its morphological, genetic, geographical, and ecological characteristics for taxonomic validation^2^. Further, the study recommends the utilization of diverse computational approaches in identifying species through DNA analysis. This is crucial for assessing whether the observed genetic variation in the species is a result of formation of cryptic species or the divergence can be attributed to misidentification stemming from the reliance on a single computational method. The study specifically addresses the freshwater fishes of the river Beas that hold commercial importance to the local livelihood and protein requirements. During a survey conducted at commercial landing stations near Beas River, we identified one endangered species, six vulnerable species, and one near threatened species. Of particular concern is the endangered species *Tor putitora,* commonly known as Golden mahseer, which holds a high value both the table and in the commercial markets. In India, the indiscriminate fishing of Golden mahseer persist due to lack of market control and specific conservation measures, despite the species’ ecological and economic significance^38^. The Department of Fisheries in Himachal Pradesh, India has implemented legal measures to restrict the fishing of Golden mahseer during breeding seasons. With a well-executed grassroots strategic plan, these measures have the potential to effectively conserve the Mahseer population. Despite various conservation efforts, several vulnerable species including *Cyprinus carpio, Cirrhinus cirrhosus, Schizothorax richardsonii, Schizothorax plagiostomus, Wallago attu*, and *Bagarius bagarius* continue to be exploited for commercial purposes in the Beas River. The wild form of *C. carpio* is classified as vulnerable, yet it is widely considered highly invasive and has been domesticated and introduced globally^39^. The decline of *C. cirrhosis*, *W. attu*, and *B. bagarius* in the Indo-Ganges River basin is linked to various factors, including habitat degradation caused by environmental changes, human interventions disrupting spawning and feeding, depletion of aquatic environments, especially for brood fish, and insufficient management of fish catchment^40–42^. *S. richardsonii* along with several other cold-water fish species, has significantly declined and is categorized as vulnerable on the IUCN red list^43^. The irreversible alterations in their native population structure and increase in unnatural deaths can be attributed to intense human intervention and the introduction of exotic species^44^. In the cold waters fish of the Beas, *S. plagiostomus*, a fish known as “game-food fish” is commercially exploited by locals, despite its vulnerable status ^45^. The majority of identified species are native to India, while three species; *Cyprinus carpio, Oreochromis niloticus,* and *Salmo trutta fario* are introduced species. Notably, the globally cultivated exotic fish *Oreochromis niloticus,* has been reported in the Beas River for the first time. Although most of the introduced species seem to have a minimal impact on native biodiversity, their global spread poses a potential threat of becoming highly invasive in certain regions which may lead to significant negative impacts on some of the native species, and eradication of their genetic resources^57^. India has witnessed the consequences of climate fluctuation and dam constructions on freshwater river systems, evident in changes to the geographic distribution, breeding season, life cycle, and physiological behavior of inland fishes^47,48^. The Pong Dam, constructed on  the Beas River, functions as both a wetland and a wildlife sanctuary. However, the excessive utilization of ecosystem services has resulted in the spread of invasive species, obstacles to fish migration, and decline in fish diversity. Additionally, overfishing and illegal trade of fishes by the local population are adversely impacting fish biodiversity in the Beas River^58,59^. Furthermore, the contamination of water resources from chemical effluent leakage from sugar mill has resulted to numerous fish mortality^60^. The uncontrolled discharge of municipal solid waste and sewage into the Beas River and its tributaries poses a significant concern. To effectively manage the aquatic biodiversity of freshwater fishes, it is crucial to monitor waste disposal in the river system and regulate the overfishing of fish fauna, especially for endangered, vulnerable, threatened, and rare species.

## Conclusion

Despite the significant importance of freshwater fish to biodiversity and as a natural resource, there is still limited information on the genetics of most species. Comprehending the contribution of diverse species to ecological systems and effectively conserving biodiversity demands a profound understanding of species diversity and the composition of distinct geographic regions. DNA barcoding extends beyond species identification, finding application in various areas such as food authentication, identification of cryptic species, conservation biology, evolutionary biology, and providing essential genetic insights for assessing genetic divergence within a species. Moreover, the ichthyofaunal population is undergoing a reduction in genetic diversity due to widespread release of allochthonous fish which may lead to a significant decline or even disappearance of native populations. DNA barcoding holds the potential to play a crucial role in formulating effective conservation plans for wild fishes, ensuring the maintenance of their genetic diversity. To establish strategies and practical measures that ensure the proper identification and sustainable use of aquatic resources of freshwater river systems, we recommend integrating DNA barcoding with different species delimitation methods and accurate geographic distribution information. We further propose incorporating both morphological identification and DNA barcoding as an integral component of regular monitoring practices for genetic diversity, a vital tool for conservation of biodiversity and their genetic resources.

## Materials and methods

### Ethical statement

In the current study, fish sampling was conducted at all commercial landing stations adjacent to Beas River. Prior to sampling, the necessary approval was obtained from licensing authority of Himachal Pradesh State Biodiversity Board, Himachal Pradesh, India vide letter no. HIMCOSTE/HPSBB/2019/358 dated 05/12/2019. For this study, we exclusively utilized non-invasive tissue samples for DNA barcoding, which do not require any animal ethical clearance. Nevertheless, all procedures for handling the fish samples were carried out in accordance with the guidelines established by the Institutional Animal Ethical Committee (IAEC).

### Sample collection

Fish specimens were obtained using two methods. The first method involved capturing fish using casting nets and local punga jaal (hand nets). The method involved catching fish with the help of local fishermen, taking scales or fins for non-invasive sampling, and then releasing them back into the water. The second method involved collecting tissue samples from fish at nearby landing centers of river Beas. A total of 320 fish specimens were collected from February 2020 to September 2022 from fifteen sites within the river system (Fig. 1). The river system was divided into three major areas. The first area, upstream of the Beas River system comprised of three locations in Himachal Pradesh namely Gammon bridge (stretch along Ramshila road, Manali), Old Manali road (Siyal), and Bajaura. The second area, the midstream in the river system in Kangra district of Himachal Pradesh included Khatiyar fish landing station, Barnali fish landing station, Gajj, Jwali Khad, Pong dam reservoir, and Sthana landing station. The third location was in Punjab, downstream of the river system and constituted Kiriyan, Talwandi, Jogyawal Maza walla, the Harike wetland landing station, Beas mand area, and Kapurthala landing station. Initially, all specimens were identified up to genus level based on morphological characteristics using identification keys ^61^. Tissue and fin clippings from the specimens were preserved in absolute ethanol and transported to the laboratory. Samples were then stored at − 20 °C until DNA isolation.

### DNA extraction and COI amplification

Total genomic DNA was isolated from muscle tissue or fin clipping of 203 samples using the standard phenol–chloroform protocol^62^. A NanoDrop 1000 Spectrophotometer was used to quantify the isolated DNA, followed by visualization in 0.8% agarose gel electrophoresis. The amplification of universal COI primers FishF1 (5′-TCAACCAACCACAAAGACATTGGCAC-3′) and FishR1 (5′-TAGACTTCTGGGTGGCCAAAGAATCA-3′) was performed using a Polymerase chain reaction (PCR)^7^. The PCR reaction mixture (50 µl) containing 1.5 mM 10× buffer, 25 mM MgCl_2_, 10 mM dNTPs, 1.5 µl DMSO, 3U Taq polymerase, 2 pmol of each primer, 2 µl (~ 50 ng) DNA template and deionized water. The amplification protocol included an initial denaturation step at 95 °C for 5 min, followed by 35 cycles of denaturation at 95 °C for 30 s, annealing at 58 °C for 30 s, extension at 72 °C for 45 s, and final extension at 72 °C for 7 min. Amplified products were separated on a 1.5% (w/v) agarose gel by loading 50 µl of PCR product and 3 µl loading dye. The bands of amplification were visualized under UV light with ethidium bromide and eluted using the QIAquick Gel Extraction Kit by Qiagen. The eluted PCR products were sent to Apical Scientific Laboratory (1st Base, Selangor, Malaysia) for Sanger sequencing.

### Sequence-based identification and genetic data analysis

Sequence chromatograms were examined for quality using Sequencing Analysis software v5.1.1. The dataset of 203 sequences had an average length of 640 bp. Sequences were identified using NCBI Blast nucleotide and BOLD (Barcode of Life Data System) identification engine. All sequences were submitted to the BOLD (https://www.boldsystems.org/) database under the project “DNA barcoding of Fishes of Beas River” (code: BEAS) and GenBank with accession numbers (OR148039—OR148241). To estimate genetic divergence and phylogenetic relationships, 485 sequences belonging to the same species with sampling sites from India were retrieved from BOLD. The coordinates of sampling locations and corresponding specimen information for each sequence is given in Supplementary Table S1. A total of 688 sequences representing 43 species were analyzed to calculate genetic divergence at various taxonomic levels using Kimura two-parameter model (K2P)^63^ in BOLD v4^64^. BioEdit (https://bioedit.software.informer.com/) was used to assemble and align all the sequences. DnaSP v5^65^ was used to estimate haplotype frequency and diversity within different species. Nexus files were created from aligned sequences in DnaSP v5 software to build a median joining haplotype network and to determine the frequency of haplotypes in various regions. PopART^66^ was then used to construct the median-joining network of haplotypes using default parameters. A phylogenetic tree was constructed among sequences using the neighbor-joining (NJ) approach with the K2P model and 1000 bootstrap replicates in MEGA X (https://www.megasoftware.net/).

For species delimitation, Automatic Barcode Gap Discovery (ABGD) tool (https://bioinfo.mnhn.fr/abi/public/abgd/abgdweb.html)^67^ was used with K2P model, Transition/transversion ratio (TS/TV) = 2.0, relative gap width (X) = 1.0, Prior Intra-species divergence from Pmin = 0.001 to Pmax = 0.1, and distance distribution (Nb bins) = 20. Phylogenetic relationships were reconstructed using Bayesian Inference (BI) analysis in MrBayes 3.2.7^68^. The dataset was run with gamma-distribution rate variation, invariable positions (GTR + G + I), and six substitution sites (nst = 6). MrBayes was run with Monte Carlo Markov chains (MCMC) for 20,000,000 generations, sampling every 10,000 generations, and a chain temperature of 0.2. The initial 25 percent of the trees were burned in and the resulting tree file was visualized using FigTree v1.4.4 (http://tree.bio.ed.ac.uk/).

### Supplementary Information

Supplementary Figure 2.

Supplementary Information 2.

Supplementary Table S1.

## Data Availability

Sequence data and collection information have been made available on BOLD (www.boldsystems.org), Project code: BEAS. Sequences are also deposited in GenBank (Accession numbers OR148039—OR148241). The overall dataset of sequences analyzed in the study has been made publicly visible on BOLD (Dataset Code: DS-INTF).
