# Supplementary figures and images for: A comprehensive DNA barcoding of Indian freshwater fishes of the Indus River system, Beas
Source: Sci Rep. 2024 Feb 2;14:2763. doi: 10.1038/s41598-024-52519-0 (PMC10837433; doi:10.1038/s41598-024-52519-0)

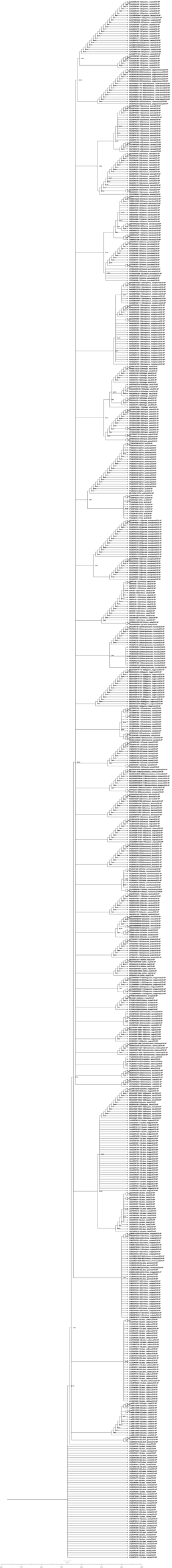

Supplement: Supplementary file 1 — Supplementary Figure 2. [file 41598_2024_52519_MOESM1_ESM.pdf]
