# Supplementary material for: A comprehensive DNA barcoding of Indian freshwater fishes of the Indus River system, Beas
Source: Sci Rep. 2024 Feb 2;14:2763. doi: 10.1038/s41598-024-52519-0 (PMC10837433; doi:10.1038/s41598-024-52519-0)
Supplement: Supplementary file 2 — Supplementary Information 2. [file 41598_2024_52519_MOESM2_ESM.docx]

**Supporting document**

**A Comprehensive DNA Barcoding of Indian Freshwater Fishes of the Indus River System, Beas**

Sonakshi Modeel^1^, Ram Krishan Negi^1^*, Monika Sharma^1^, Padma Dolkar^1^, Sheetal Yadav^1^, Sneha Siwach^1^, Pankaj Yadav^1^, and Tarana Negi^2^

^1^ Fish Molecular Biology Lab, Department of Zoology, University of Delhi, North Campus, Delhi 110007, India

^2^ Department of Zoology, Govt. College, Bahadurgarh, District Jhajjar, (HR), India

*Corresponding Author

Corresponding Author Email: [negigurukul@gmail.com](mailto:negigurukul@gmail.com)

Corresponding Author (ORCID ID): https://orcid.org/0000-0002-5753-8376

**Table S1: Project code, sample IDs, taxon, and collection data of sequences analysed in the study.**

Provided as separate supplementary material in the form of a spreadsheet (.xlsx).

**Table S2: Table representing the mean and maximum intra-specific values compared to the nearest neighbor distance.**

| **Order** | **Family** | **Species** | **Mean Intra-Sp** | **Max Intra-Sp** | **Nearest Species** | **Nearest Neighbour (NN)** | **Distance to NN** |
| --- | --- | --- | --- | --- | --- | --- | --- |
| Anabantiformes | Channidae | *Channa marulius* | 2.47 | 8.96 | *Channa punctata* | GDK355-13 | 17.29 |
| Anabantiformes | Channidae | *Channa punctata* | 0.63 | 1.46 | *Channa marulius* | NRSC049-12 | 17.29 |
| Anabantiformes | Nandidae | *Nandus nandus* | 0.48 | 1.63 | *Mastacembelus armatus* | BEAS261-22 | 21.73 |
| Beloniformes | Belonidae | *Xenentodon cancila* | 0.74 | 2.08 | *Chanda nama* | BEAS336-22 | 19.24 |
| Cichliformes | Cichlidae | *Oreochromis niloticus* | 0.03 | 0.16 | *Tor putitora* | BEAS304-22 | 19.66 |
| Clupeiformes | Clupeidae | *Gudusia chapra* | 0.13 | 0.49 | *Labeo catla* | BEAS397-22 | 21.69 |
| Cypriniformes | Cyprinidae | *Bangana dero* | 0 | 0 | *Labeo boggut* | BEAS244-22 | 0 |
| Cypriniformes | Cyprinidae | *Barilius vagra* | 0.99 | 3.07 | *Tariqilabeo adiscus* | BEAS333-22 | 17.5 |
| Cypriniformes | Cyprinidae | *Cabdio morar* | 0.6 | 1.31 | *Labeo boggut* | GDK736-13 | 14.68 |
| Cypriniformes | Cyprinidae | *Chagunius chagunio* | 0.6 | 1.14 | *Schizothorax plagiostomus* | BEAS364-22 | 16.35 |
| Cypriniformes | Cyprinidae | *Cirrhinus cirrhosus* | 0.06 | 0.16 | *Cirrhinus mrigala* | GDK344-13 | 0 |
| Cypriniformes | Cyprinidae | *Cirrhinus mrigala* | 0.36 | 1.68 | *Cirrhinus cirrhosus* | GBGC4256-08 | 0 |
| Cypriniformes | Cyprinidae | *Cirrhinus reba* | 0.37 | 3.2 | *Labeo bata* | ANGEN264-15 | 0.16 |
| Cypriniformes | Cyprinidae | *Cyprinus carpio* | 0.25 | 0.9 | *Labeo bata* | FNWG015-16 | 9.06 |
| Cypriniformes | Cyprinidae | *Esomus danrica* | 0.35 | 1.56 | *Cirrhinus reba* | DBFN157-11 | 16.01 |
| Cypriniformes | Cyprinidae | *Labeo bata* | 1.96 | 13.96 | *Labeo boggut* | BEAS244-22 | 0 |
| Cypriniformes | Cyprinidae | *Labeo boggut* | 0.33 | 1.27 | *Bangana dero* | ANGBF18637-19 | 0 |
| Cypriniformes | Cyprinidae | *Labeo calbasu* | 0.2 | 1.09 | *Labeo gonius* | BEAS279-22 | 4.65 |
| Cypriniformes | Cyprinidae | *Labeo catla* | 0.15 | 0.94 | *Labeo rohita* | BEAS401-22 | 5.85 |
| Cypriniformes | Cyprinidae | *Labeo gonius* | 5.7 | 11.85 | *Cirrhinus mrigala* | GDK344-13 | 0 |
| Cypriniformes | Cyprinidae | *Labeo rohita* | 0.11 | 1.01 | *Labeo gonius* | BEAS279-22 | 5.48 |
| Cypriniformes | Cyprinidae | *Osteobrama cotio* | 5.62 | 14.66 | *Tor tor* | GDK032-11 | 12.66 |
| Cypriniformes | Cyprinidae | *Pethia conchonius* | 5.21 | 15.42 | *Tariqilabeo latius* | BEAS337-22 | 13.79 |
| Cypriniformes | Cyprinidae | *Salmostoma bacaila* | 0.42 | 0.64 | *Salmostoma phulo* | BEAS285-22 | 15.5 |
| Cypriniformes | Cyprinidae | *Salmostoma phulo* | 0.29 | 0.69 | *Salmostoma bacaila* | BEAS289-22 | 15.5 |
| Cypriniformes | Cyprinidae | *Schizothorax plagiostomus* | 0.1 | 0.34 | *Schizothorax richardsonii* | ANGBF21184-19 | 0 |
| Cypriniformes | Cyprinidae | *Schizothorax richardsonii* | 0.14 | 0.63 | *Schizothorax plagiostomus* | BEAS356-22 | 0 |
| Cypriniformes | Cyprinidae | *Systomus sarana* | 1.22 | 2.71 | *Labeo rohita* | BEAS390-22 | 11.98 |
| Cypriniformes | Cyprinidae | *Tariqilabeo adiscus* | 0.11 | 0.34 | *Tariqilabeo latius* | BEAS337-22 | 0 |
| Cypriniformes | Cyprinidae | *Tariqilabeo latius* | 0.33 | 0.33 | *Tariqilabeo adiscus* | BEAS317-22 | 0 |
| Cypriniformes | Cyprinidae | *Tor putitora* | 0.07 | 0.33 | *Tor tor* | BEAS410-22 | 0 |
| Cypriniformes | Cyprinidae | *Tor tor* | 1.07 | 3.05 | *Tor putitora* | BEAS319-22 | 0 |
| Osteoglossiformes | Notopteridae | *Notopterus notopterus* | 0.65 | 2.65 | *Mastacembelus armatus* | FNWG104-16 | 19.02 |
| Ovalentaria | Ambassidae | *Chanda nama* | 4.15 | 13.93 | *Xenentodon cancila* | BEAS282-22 | 19.24 |
| Salmoniformes | Salmonidae | *Salmo trutta* | 0.09 | 0.33 | *Channa marulius* | BEAS407-22 | 19.74 |
| Siluriformes | Bagridae | *Sperata seenghala* | 0.7 | 2.4 | *Clupisoma prateri* | BEAS214-22 | 17.2 |
| Siluriformes | Heteropneustidae | *Heteropneustes fossilis* | 0.35 | 0.99 | *Clupisoma prateri* | GDK617-13 | 16.38 |
| Siluriformes | Ritidae | *Rita rita* | 0.32 | 1.31 | *Clupisoma prateri* | BEAS214-22 | 16.51 |
| Siluriformes | Schilbeidae | *Clupisoma prateri* | 2.14 | 9.5 | *Heteropneustes fossilis* | BEAS276-22 | 16.38 |
| Siluriformes | Siluridae | *Ompok pabo* | 4.71 | 15.45 | *Wallago attu* | GDK752-13 | 15.67 |
| Siluriformes | Siluridae | *Wallago attu* | 0.46 | 1.08 | *Ompok pabo* | GBGC8964-09 | 15.67 |
| Siluriformes | Sisoridae | *Bagarius bagarius* | 0.11 | 0.99 | *Clupisoma prateri* | GDK391-13 | 16.8 |
| Synbranchiformes | Mastacembelidae | *Mastacembelus armatus* | 1.5 | 4.73 | *Channa marulius* | NRSC049-12 | 18.53 |

**
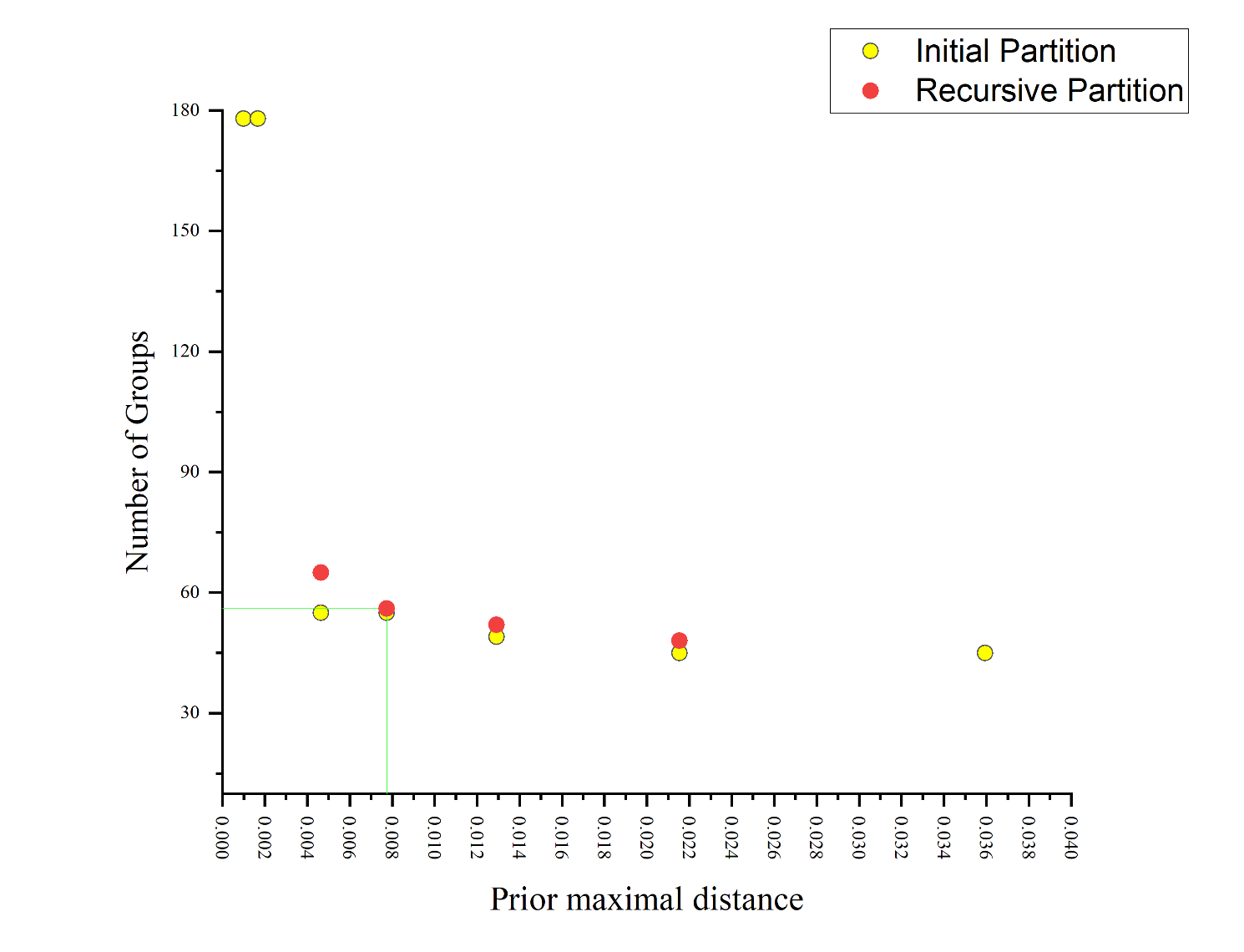
**

**Fig. S1: Automatic Barcode Gap Discovery (ABGD) analysis: The number of groups inside the partitions as a function of the prior limit between and within species divergence. The yellow color denotes the initial partition, the red color denotes the recursive partition and the dotted blue line represents the threshold value of the prior maximal distance for partition (P) = 0.0077 for species delimitation.**

**Fig. S2: K2P genetic divergence-based Neighbor-joining tree of 688 COI sequences of River Beas representing 43 species. Bootstrap values are indicated (1000 replications).**

Provided as separate supplementary material in the form of a pdf document.
